# Main Metabolites of *Pseudomonas aeruginosa*: A Study of Electrochemical Properties

**DOI:** 10.3390/s22134694

**Published:** 2022-06-22

**Authors:** Sylvia Schneider, Jörg Ettenauer, Ildiko-Julia Pap, Christoph Aspöck, Julia Walochnik, Martin Brandl

**Affiliations:** 1 Department for Integrated Sensor Systems, University for Continuing Education Krems, 3500 Krems, Austria; joerg.ettenauer@boku.ac.at (J.E.); martin.brandl@donau-uni.ac.at (M.B.); 2 Department of Biotechnology, University of Natural Resources and Life Sciences, 1190 Vienna, Austria; 3 Clinical Institute for Hygiene and Microbiology, University Hospital St. Poelten, 3100 Sankt Poelten, Austria; ildiko-julia.pap@stpoelten.lknoe.at (I.-J.P.); christoph.aspoeck@stpoelten.lknoe.at (C.A.); 4 Karl Landsteiner University of Health Sciences, 3500 Krems, Austria; 5 Institute of Specific Prophylaxis and Tropical Medicine, Medical University of Vienna, 1090 Vienna, Austria; julia.walochnik@meduniwien.ac.at

**Keywords:** electrochemical detection, *Pseudomonas aeruginosa*, metabolite, pyocyanin, pyochelin, PQS, HQNO, HHQ, screen-printed electrode

## Abstract

*Pseudomonas aeruginosa* is a ubiquitously distributed soil and water bacterium and is considered an opportunistic pathogen in hospitals. In cystic fibrosis patients, for example, infections with *P. aeruginosa* can be severe and often lead to chronic or even fatal pneumonia. Therefore, rapid detection and further identification are of major importance in hospital hygiene and infection control. This work shows the electrochemical properties of five *P. aeruginosa* key metabolites considering their potential use as specific signaling agents in an electrochemical sensor system. The pure solutes of pyocyanin (PYO), *Pseudomonas* quinolone signal (PQS), pyochelin (PCH), 2-heptyl-4-hydroxyquinoline (HHQ), and 2-heptyl-4-hydroxyquinoline N-oxide (HQNO) were analyzed by different electrochemical techniques (cyclic and square wave voltammetry) and measured using a Gamry Reference 600+ potentiostat. Screen-printed electrodes (DropSens DRP110; carbon working and counter, silver reference electrode) were used to determine signal specificities, detection limits, as well as pH dependencies of the substances. All of the compounds were electrochemically inducible with well-separated oxidation and/or reduction peaks at specific peak potentials relative to the reference electrode. Additionally, all analytes exhibited linear concentration dependency in ranges classically reported in the literature. The demonstration of these properties is a promising step toward direct multiplexed detection of *P. aeruginosa* in environmental and clinical samples and thus, can make a significant contribution to public health and safety.

## 1. Introduction

*Pseudomonas aeruginosa* is a gram-negative bacterium and opportunistic human pathogen that has received enormous attention in recent decades. In 2011, it was first categorized as a top priority infectious agent by the Robert Koch Institute, Germany [1]. Additionally, in 2013 *P. aeruginosa* was classified as a serious threat in the CDC antibiotic resistance threat report [2]. The reasons for these classifications are manifold. *P. aeruginosa* can pose a major health risk in immunocompromised individuals or persons with predisposing factors, underlying diseases (e.g., cystic fibrosis), or physical damages (e.g., wounds or traumatic burns). Diseases caused by *P. aeruginosa* range from eye and ear, chronic respiratory tract, and wound infections to gastrointestinal tract infections, as well as blood infections with various sepsis syndromes [3,4]. Moreover, *P. aeruginosa* is considered one of the most common causes of healthcare-associated infections. Between 2006 and 2017, it was the cause of 7–9% of reported nosocomial infections in Europe and the United States [5,6,7,8]. The bacterium has a versatile energy metabolism and low nutritional requirements. It can adapt to conditions not tolerated by other organisms [9,10] and also has a ubiquitous distribution. Consequently, the reservoir for potential infection of at-risk individuals is relatively large. In addition, *P. aeruginosa* strains can grow in a biofilm form that renders the cells insensitive to antibacterial agents such as disinfectants or host defense mechanisms [3]. In addition, many strains of *P. aeruginosa* have become resistant to a variety of antibiotics commonly used in clinical practice [11,12].

Suitable precautions must therefore be taken, particularly in medical institutions, to avoid infections in patients who are already at risk and weakened. This includes not only careful hand hygiene practices but also, if necessary, the control of potentially contaminated sources. On the other hand, when a patient is already infected, an effective treatment is required. In all instances, the further identification of *P. aeruginosa* is necessary, which is usually achieved by classical cultivation [13,14], molecular methods [15,16], or by MALDI-TOF (matrix-assisted laser desorption/ionization—time of flight) techniques [17,18]. However, all of these methods are time-consuming and/or require expensive equipment and trained personnel. In recent years, with the advancement of microelectronic technologies, electrochemical sensor systems have become increasingly attractive for microbiological issues [19]. The relatively simple setup, high sensitivity, and speed of operation make them very attractive for point-of-care testing in clinical settings and large-scale screenings.

In previous studies, authors focused on the electrochemical detection of *P. aeruginosa* using its blue pigment and main virulence factor, pyocyanin (PYO) (summarized by Alatraktchi et al. [20]). Pyocyanin is produced exclusively [21,22] by 94–100% of *P. aeruginosa* strains [22,23,24] and is thus a suitable target for the development of a specific detection method. It is redox-active [25] and can be detected electrochemically, for example, in culture supernatants of clinical isolates [24] or in chronic wound exudates [26]. To date, however, there is only one work that addresses the electrochemical behavior of pyocyanin itself in detail [27]. Other metabolites of interest, such as the *P. aeruginosa* quinolones PQS (*Pseudomonas* quinolone signal or 2-heptyl-3-hydroxy-4-quinolone) and HHQ (2-heptyl-4-hydroxyquinoline), are less well studied electrochemically. For example, some recent contributions on this topic were made by Zhou et al. [28], Buzid et al. [29], and Oziat et al. [27]. In addition, some metabolites have not been investigated at all for their electrochemical properties, such as in the case of the further quinolone HQNO (2-heptyl-4-hydroxyquinoline N-oxide) and the siderophore pyochelin (PCH).

In this work, we extensively characterized the above molecules of the *P. aeruginosa* secretome on screen-printed electrodes (SPEs) regarding their potential use as specific signaling agents in an electrochemical sensor system. One aspect to consider was that electrochemical tests are strongly influenced by the pH of sample solutions. If it changes due to bacterial growth, knowing about the pH dependency of the chemical reactions would allow for the prediction of the signals. In addition, the pH of clinical samples can vary widely. For example, chronic wounds become alkaline over time (pH 7.15 to 8.9) [30], whereas the airways of cystic fibrosis patients are abnormally acidic (mean of pH 5.3 in patients with exacerbation) [31]. Therefore, the effects of the pHs of the electrolytes on peak potentials and peak currents were also investigated using different electrochemical techniques—cyclic (CV) and square wave voltammetry (SWV). Additionally, the limits of detection (LOD) of the metabolites depending on the buffers and media in which *P. aeruginosa* may be grown are reported.

In a fast and possibly automated sensor system, the detection of *P. aeruginosa* should be based on the signal from more than one biomarker to increase specificity and sensitivity. The simultaneous detection of multiple molecules could provide a comprehensive and reliable signal. This is the only way to allow for the accurate identification of *P. aeruginosa* in environmental or clinical samples—even in those without the blue coloration of PYO. Therefore, the exact information about the redox reactions of the molecules is a prerequisite for compound recognition and thus for rapid indications of possible contaminations with the pathogen. For this reason, we defined the peak situations of not only one but five major metabolites released by the bacterium. The more signals can be assigned to distinctive substances, the better the detection of *P. aeruginosa* can be enabled in a well-functioning and safe sensor system. The present work thus gives a complete and extensive characterization of these molecules, their voltammetric behavior under different conditions, and a comparison of the signals recorded on small SPEs. The results of this study should help to provide a basis for a reliable and sensitive sensor system for the detection of *P. aeruginosa*.

## 2. Materials and Methods

### 2.1. Chemicals and Reagents

The chemicals were purchased from Sigma-Aldrich, Saint Louis, MO, USA (2-heptyl-4-hydroxyquinoline (HHQ), *Pseudomonas* quinolone signal (PQS), pyocyanin (PYO)), from Cayman Chemical, Ann Arbor, MI, USA (2-heptyl-4-hydroxyquinoline N-oxide (HQNO)), and from Toronto Research Chemicals, Toronto, Canada (pyochelin (PCH)), respectively. They were used at the highest purity grade and without any further purification. Stock solutions were prepared in acetonitrile (ACN) in the following concentrations: 6.6 mM HHQ, 12.9 mM HQNO, 3.4 mM PCH, 4.3 mM PQS, and 8 mM PYO. Stocks were aliquoted, stored at −20 °C, and further diluted in buffers or media just before testing. Buffers with a wide pH range (pH 2 to 10.8) were prepared with distilled, ultra-pure water containing sodium chloride (170 mM) and 10 mM buffer reagent according to the pH scale. For buffers of pH 2 to 4.5, mixtures of acetic acid and sodium acetate, and for pH 5.7 to 8, mixtures of sodium phosphate dibasic and sodium phosphate monobasic were prepared. For buffers of pH 9.1 to 10.8, mixtures of sodium carbonate and sodium hydrogen carbonate were used. LB broth (Lennox) was prepared according to the manufacturer’s information (Carl Roth, Karlsruhe, Germany), and *Pseudomonas* medium without (PM) or with selective additives (0.2 g/L cetrimide and 0.015 g/L nalidixic acid) (PMCN) was prepared according to [14]. All of the buffers or media were sterile filtered or autoclaved.

### 2.2. Electrochemical Sensors

All of the electrochemical measurements were performed with disposable, screen-printed electrodes (SPE) (DRP-110, Metrohm DropSens, Oviedo, Spain). The working electrode and counter electrode were made of carbon, whereas the reference electrode was silver (Ag). For each scan, we applied 100 µL of the test solution to a new electrode. All of the electrochemical tests were performed with a Gamry potentiostat Reference 600+ equipped with Gamry Framework software (Gamry Instruments, Warminster, PA, USA) using either CV or SWV. In general, the voltammetric analyses were performed in a potential range from −800 to 1100 mV, which represents the measurable potential window for these SPEs (without the current peaks of water electrolysis). After the determination of the individual peak positions, the measurement windows were reduced in size, depending on the substance, to allow a more precise characterization (cf. scales of the graphs in the Results section). The correlation of peak current with scan rate was determined by varying the scan rate from 10 to 500 mV/s during CV; otherwise, the usual CV scan rate was 100 mV/s (unless otherwise specified). The general parameters for SWV were a step size of 0.5 mV, an amplitude of 50 mV, and a frequency of 5 Hz (unless otherwise stated). Data editing and calculation were performed with Gamry Echem Analyst (Gamry Instruments, Warminster, PA, USA). The statistical analyses and graphical processing were conducted in GraphPad Prism 9.1 (GraphPad, San Diego, CA, USA). All of the graphs and data are based on laboratory experiments and measurements with the potentiostat. The results (potentials and currents) without further indication are referred to the Ag reference system.

## 3. Results and Discussion

The properties of the studied metabolites are given in the following section. Different electrochemical techniques (CV and SWV) were carried out on screen-printed electrodes (SPE).

### 3.1. Electrochemical Properties of Pseudomonas Quinolone Signal (PQS)

**Figure 1:**
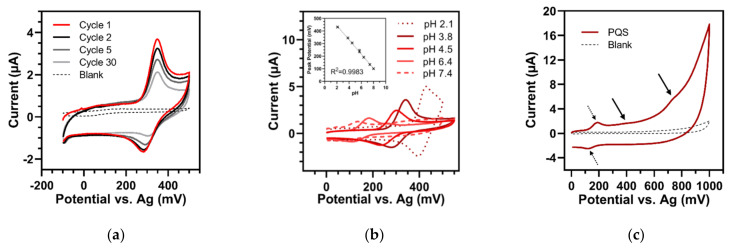
Cyclic voltammetry of 250 µM PQS (100 mV/s). (**a**) First 30 cycles in buffer of pH 3.8. The intensity of the redox reaction decreased from the first (red) to the repeated cycles (black to light grey). Blank = pH 3.8 buffer. (**b**) Dependency of peak potential on pH of the buffer solution. Insert: Linear regression of oxidation peak potential versus pH in the whole measurable spectrum (pH 2.1 to 8.0). Error bars represent the standard deviations of three replicates. (**c**) Performance of PQS in wide measurement range from 0 to 1000 mV (pH 6.4). Arrows show the quasi-reversible redox couple (at +191/+114 mV) (dashed) and the additional oxidation waves at approx. 410 and 740 mV (full). Blank = pH 6.4 buffer.

Cyclic voltammetry allowed for the confirmation of one main redox couple for PQS in an aqueous buffer system. An example of a typical CV scan is given in Figure 1a, where in a buffer of pH 3.8, the oxidation and reduction peaks were found at 348 mV and 282 mV, respectively. However, at all measurable pH levels, the amplitudes of the reported couple dropped between the first and subsequent cycles. In addition, the intensities of the reduction peaks were consistently lower than those of the oxidation peaks. For the given example in a pH 3.8 buffer, the oxidation and reduction peaks had a ratio of 0.36 (at 100 mV/s) and decreased by 17% and 27%, respectively, from the first to the second cycle. This indicated that the electrochemical reaction was not fully reversible, possibly due to the formation of a non-reversible species.

Otherwise, when the scan rate was increased, the oxidation and reduction peak currents showed no linear dependency on the square root of the scan rate nor on the scan rate (Figure S1). Moreover, the peak-to-peak separation (ΔE_p_) at pH 6.4 and at 100 mV/s had an average of 77 mV. On the one hand, this could indicate that not more than one electron was involved in the redox reaction. However, otherwise, the enlarged ΔE_p_ compared to the theoretical 59 mV (given by the Nernst equation) suggests a sluggish reaction that was somehow hindered on the electrode surface. Additionally, the separation increased rapidly as the scan rate increased. In a buffer of pH 6.4, ΔE_p_ was 130 mV at a scan rate of 500 mV/s. All of these observations pointed to the fact that PQS underwent an electrochemical quasi-reversible redox reaction in this system. These findings were consistent with the work of Oziat et al. [27], who also suggested a quasi-reversible redox reaction of PQS, except on Glassy carbon (GC) working electrodes with an Ag/AgCl reference system.

Furthermore, the peak potential of PQS shifted linearly with buffer pH (pH 2.1 to 8.0) and with a slope of −56.9 ± 1.1 mV/pH unit (Figure 1b). This result was very similar to the theoretical Nernstian −59 mV/pH unit (at 25 °C) for redox reactions exchanging the same number of protons and electrons. In accordance with [29], we found that the amplitude of the peak decreased with elevated pH and finally disappeared completely at a pH higher than 8.0. This also revealed the transfer of protons during the reaction. The electrocatalytic oxidation became less favorable at higher pH, resulting in lower peak currents.

In addition to this aforementioned quasi-reversible redox pair, several non-reversible oxidation waves of PQS in pH 6.4 buffer could be seen when pushing the CV scanning potential towards higher values (up to the maximum of the measurable potential window of about 1000 mV) (Figure 1c). Nevertheless, these waves were poorly reproducible in shape and intensity from one experiment to another. Oziat et al. [27] also observed a total of three redox reactions in acetonitrile on the GC electrode, which may correspond to the oxidation reactions reported here. In agreement with [32], they suggested the exchange of only two electrons and two protons, which could, however, originate from two possible tautomeric forms of the molecule. This could eventually result in three redox reactions during CV. Our observations confirmed the redox behavior proposed by Oziat et al. [27].

### 3.2. Electrochemical Properties of 2-heptyl-4-hydroxyquinoline (HHQ)

**Figure 2:**
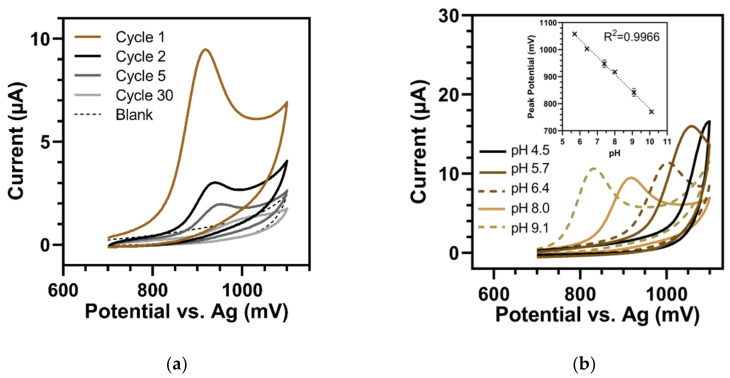
Cyclic voltammetry of 125 µM HHQ (100 mV/s). (**a**) First 30 cycles in buffer of pH 8.0. In the first cycle (brown), one defined oxidation peak at 918 mV occurred. In the following cycles (black to light grey), the oxidation peaks rapidly dropped. Blank = pH 8.0 buffer. (**b**) Dependency of HHQ on pH of the buffer solution. In acid buffers (≤pH 5.0), no oxidation peaks could be seen, i.e., could not be separated from the peaks of water electrolysis. Insert: Shift in oxidation peak potential versus pH (pH 5.7 to 10.1). Error bars represent the standard deviations of three replicates.

Voltammetric measurements of HHQ showed that its oxidation potentials were highly anodic. For example, the CV of HHQ in phosphate buffer of pH 8.0 showed one irreversible oxidation wave at a peak potential of 918 mV (Figure 2a). Moreover, the peaks of HHQ dramatically lost their intensities over repeated cycles (by 71.5% from the first to the second cycle), suggesting that the oxidation product of the irreversible process was deposited onto the electrode surface. Oziat et al. [27] also attributed the sharp decrease in the electrochemical response of HHQ after the first cycle to strong surface fouling of their GC electrode.

However, Oziat et al. [27] did not show CV measurements of HHQ at acid pH. In these ranges, we found that the oxidation reactions of HHQ shifted to high potentials where they interfered with spontaneous peaks due to water electrolysis (at about 1100 mV). In contrast to former CV analyses on boron-doped diamond electrodes, where HHQ gave an oxidation peak at +1.473 V at pH 2.0 [28], HHQ could not be detected with the present SPEs in buffers with a pH less than or equal to 5 (Figure 2b). Otherwise, in the measurable range (pH range of 5.7 to 10.1), the oxidation peak potential of HHQ decreased as a linear function of the pH. The slope of the regression curve was with −63.8 ± 1.8 mV/pH unit, similar to the Nernst value for chemical reactions that involved as many electrons as protons. Oziat et al. [27] attributed this observation to the exchange of one electron and one proton on the aromatic nitrogen of HHQ, resulting in a radical species.

### 3.3. Electrochemical Properties of Pyochelin (PCH)

**Figure 3:**
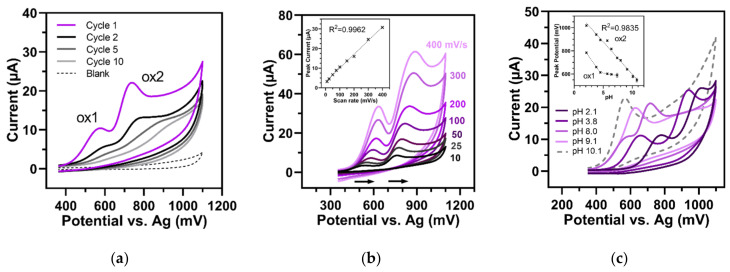
Cyclic voltammetry of 125 µM PCH (100 mV/s). (**a**) First ten cycles of CV in buffer of pH 7.4. In the first cycle (purple), two defined oxidation peaks (ox1 and ox2) at 580 mV and at 740 mV occurred, which rapidly dropped in the following cycles (black to light grey). Blank = pH 7.4 buffer. (**b**) Rise of the peak amplitudes with elevated scan rates (10 to 400 mV/s, pH 6.4). Arrows show that both peaks shifted towards higher potentials. Insert: Linear regression of peak current versus scan rate (ox1). (**c**) Dependency of peaks on buffer pH. Insert: Shift in oxidation peak potentials of ox1 and ox2 over the whole range (pH 2.1–10.8). Error bars represent the standard deviations of three measurements.

In a phosphate buffer of pH 7.4, PCH underwent purely irreversible oxidation reactions. In CV, two oxidation peaks (ox1 and ox2) were generated at high potentials of approximately 580 mV and 740 mV. With repeated scans, the intensities of the oxidation peaks heavily declined. From cycle one to cycle two, ox1 and ox2 decreased by 61% and by 51%, respectively (Figure 3a). This phenomenon was also consistent with the evolution of the oxidation peak amplitudes with the scan rate. Both peaks showed a linear increase with the scan rate, indicating the adsorption of PCH on the electrode surface. Additionally, by increasing the scan rate, a shift of both peaks towards higher potentials was detected (Figure 3b). All of these findings suggested that the oxidation of PCH led to strong surface fouling of the electrode due to the formation of a non-reactive oxidation product during the unidirectional reaction. This type of electrochemical reaction of PCH is reported here for the first time.

To determine the effect of pH on the reactions of PCH, CV was performed in a wide pH range (pH 2.1 to 10.8) (Figure 3c). The voltammetric responses of PCH between pH 2.1 and 8.0 showed two oxidation peaks (ox1 and ox2), while at higher pH values, the two peaks combined into one. This effect started with a clear peak overlapping at pH 8.0 and led to a complete union at pHs equal to or greater than 9.1 with only one intense peak. The potential of the posterior peak (ox2)—which transitioned to the combined peak at increased pH—was linearly dependent on the pH over the entire tested range (insert Figure 3c). The shift of the peak potential was with −55.9 ± 2.6 mV/pH unit, close to the theoretical value and, therefore, a sign for the exchange of the same number of protons as electrons. Conversely, the potential of the small peak (ox1) (which appeared only at pHs equal to or below 8.0) shifted dependent on the pH in ranges between pH 2.1 and 4.5. This also pointed towards the transfer of protons as well as electrons. However, at pHs above 4.5, this peak no longer evolved linearly with pH, suggesting a pure electron transfer reaction. We conclude that the substance has two oxidation systems: one in which proton and electron exchange occur over the whole tested pH range (ox2) and a second one (ox1) that may be more prone to deprotonation at neutral and slightly basic pH values, where the electrocatalytic oxidation involves only pure electrons. Our results were in accordance with the physicochemical specification of PCH by Brandel et al. [33]. They differentiated a basic phenol-thiazoline chromophore of PCH from an acid thiazolidine-4-carboxylic residue, which is easily deprotonated under basic conditions.

### 3.4. Electrochemical Properties of 2-heptyl-4-hydroxyquinoline N-oxide (HQNO)

**Figure 4:**
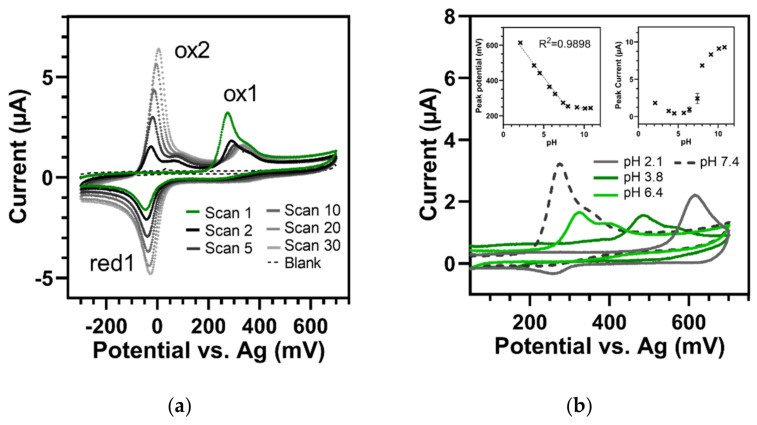
Cyclic voltammetry of 250 µM HQNO (100 mV/s). (**a**) Thirty CV cycles in buffer of pH 7.4. In the first cycle (green), one defined oxidation peak (ox1) occurred at 274 mV and one reduction peak (red1) at −46 mV. In the following cycles (black to light grey), ox1 dropped, while the second oxidation peak (ox2) at −24 mV and the shoulder at approx. 70 mV, as well as red1, increased continuously. Blank = pH 7.4 buffer. (**b**) Dependency of ox1 on buffer pH. Left insert: Shift in oxidation peak potentials versus pH (pH 2.1–10.8). Right insert: Peak current versus pH. Error bars represent standard deviations of three replicates.

A typical CV graph of HQNO is shown in Figure 4a. The first CV scan in a neutral phosphate buffer of pH 7.4 presented one irreversible oxidation peak (ox1) at 274 mV associated with an oxidation shoulder at about 350 mV and one unconnected reduction wave at −46 mV (red1). Interestingly, and unlike all other tested substances, after the first cycle, an additional oxidation peak (ox2) and shoulder appeared at −25 mV and 70 mV, respectively. The first oxidation wave (ox1) itself dropped rapidly, while the new one (ox2) and the reduction wave (red1) increased with each additional cycle. Additionally, the reduction wave (red1) and the additional ox2 occurred only when the scanning potential was pushed beyond the first oxidation wave (ox1). Limiting the potential range to −200 to 200 mV did not lead to the development of either red1 or ox2.

This phenomenon—which was observed at all measured concentrations (250–15 µM) and in buffers with diverse pH values (2.1 to 10.8)—strongly suggests that a new chemical product was generated due to the electrochemically induced irreversible reaction at ox1. This product could be reduced and reoxidized in subsequent cycles and exhibited an oxidation-to-reduction peak potential difference of 25 ± 2.2 mV on average (at 100 mV/s and pH 7.4). The value was measured over the first 10 scans after appearance and was comparable to the theoretical one of the Nernst equation (28 mV) obtained for reversible two-electron transfer systems at 25 °C. However, the ratio of reduction to oxidation peak intensities of the newly generated redox pair changed with the number of scans. After the first cycle of generation, it was 1.43; after the fourth round, it turned to about 1 and then was below 1 for further repeats, while both peaks increased steadily. We assume that first, a new product was continuously formed from the original ox1 (whose peak decreased with every cycle). After that, the generated redox couple could undergo nearly reversible reactions. Furthermore, the current responses of ox1, as well as those of the newly formed couple (ox2 and red1), varied linearly with the scan rate, indicating a slightly adsorbed species on the electrode surface (see Figure S2).

Moreover, Figure 4b displays the CV response of 250 µM HQNO over a wide pH range (2.1 to 10.8). The peak of ox1 shifted in potentials according to pH with linear behavior and a slope of −61.2 ± 2.8 mV/pH unit (between pH 2.1 and 8.0). This was consistent with the Nernstian value (−59 mV/pH at 25 °C) for a redox reaction that included as many electrons as protons. However, the shift in the peak potential was not observed at pH values above 9.1. There, no shift of ox1 occurred, suggesting a pure electron transfer reaction in which the proton had already been removed due to the alkaline pH of the sample solution. This was also consistent with the change in the height of the peak current dependent on the buffer pH (right insert in Figure 4b). At pH values above 8, oxidation obviously became more favourable, resulting in increased peaks. In this study, we were able to describe these properties of HQNO for the first time.

### 3.5. Electrochemical Properties of Pyocyanin (PYO)

**Figure 5:**
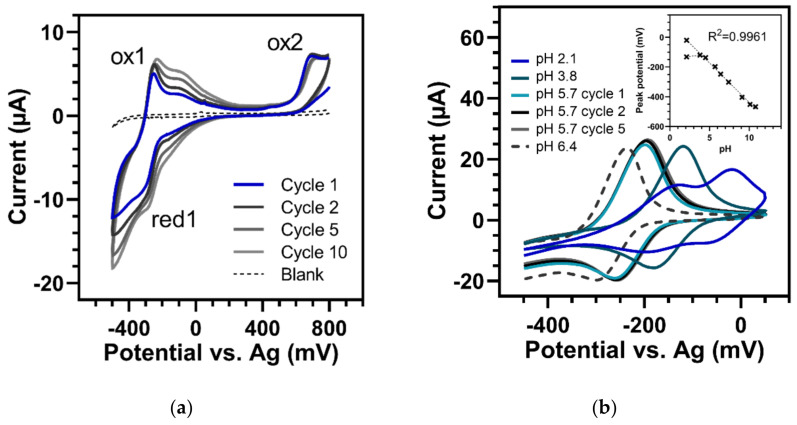
Cyclic voltammetry of PYO (100 mV/s). (**a**) First ten cycles of 125 µM PYO in buffer of pH 6.4 (potential range –500 to 800 mV). After the first cycle (blue), ox2 dropped, while the redox couple (ox1/red1) continuously increased (black to light grey). Blank = pH 6.4 buffer. (**b**) Dependency of 250 µM PYO on buffer pH. Insert: Linear regression of oxidation peak potentials according to pH (pH 2.1 to 10.8). Error bars represent the standard deviations of three measurements.

Compared to the other compounds, the virulence factor PYO has been described extensively before [27,29,34,35,36]. However, in our study, we focused on the detection of PYO on small, convenient, and disposable SPEs that require a small test volume (100 µL). On these electrodes, we could define one quasi-reversible redox wave (ox1/red1) with an oxidation peak potential of –243 mV in combination with a purely irreversible reaction at 696 mV (ox2) (in pH 6.4 buffer). Figure 5a displays the CV response of 125 µM PYO obtained at 100 mV/s when the scanning potential was pushed to high ranges (−500 to 800 mV). With repeated cycles, ox2 dropped while the redox couple at low potential steadily increased.

This phenomenon has been described before and was attributed to the presence of two possible, electrochemically-induced reactions of PYO [27,36,37]: At the negative potential, a reversible redox reaction involving two electrons and two protons (ox1/red1), and at the high positive potential (ox2), non-reversible phenolic oxidation. This is responsible for the polymerization of PYO. The polymerized form could then undergo reversible reactions at the same potentials as the lower redox couple, thus, resulting in an increase in the ox1 and red1 peaks. As shown in Figure 5a, our results on these SPEs are in accordance with previously reported studies [36,37].

Additionally, similar to [36,37], we also saw that the polymerization could be hindered when the potential range was limited to the lower peak window (−600 to 50 mV). Then, the redox pair (ox1/red1) showed reactions without an increase over several cycles (Figure 5b: repeated cycles of PYO in a buffer of pH 5.7). However, when the scan rate was varied, the currents of ox1 and red1 (125 µM PYO in buffer of pH 6.4) showed linear dependency on the scan rate (Figure S3). Furthermore, the ratio of the reduction to the oxidation peak current as well as the ΔE_p_ changed with an altered scan rate, showing that PYO underwent a quasi-reversible reaction in our system (Table S1).

Moreover, the oxidation peak potential of the redox pair decreased linearly with the buffer pH (pH 2.1 to 10.8) and with a slope of −53.2 ± 1.2 mV/pH unit (Figure 5b). This has already been explained by the transfer of two electrons as well as two protons during the reaction [27,36,37]. However, similar to [27], we saw that PYO underwent a peak separation at low pH. At pHs less than 3.8, broadening of the PYO peak started and finally led to a complete peak splitting at pH 2.1. From this point on, the potential of the lower peak did not evolve with the pH, suggesting a pure electron transfer reaction.

### 3.6. Electroanalytical Potential

**Figure 6:**
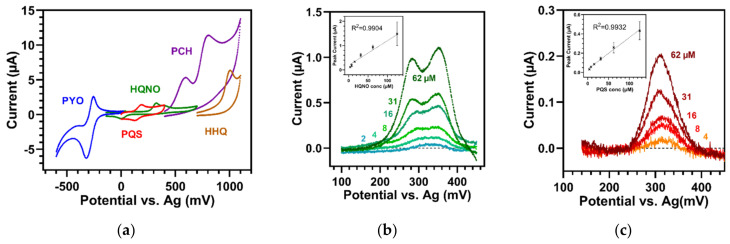
(**a**) Position of the main peaks of PYO (62 µM), PQS (250 µM), HQNO (250 µM), PCH (62 µM), and HHQ (62 µM) in CV (100 mV/s, buffer of pH 6.4). Concentrations with pronounced signals are used for comparison. (**b**,**c**) Square wave voltammograms (step size 0.5 mV, amplitude 50 mV, frequency 5 Hz) of HQNO in PMCN medium and of PQS in pH 3.8 buffer, respectively. Inserts: Calibration curves of peak currents. Error bars represent the standard deviations of triplicate measurements.

The peak situations of the five metabolites are summarized in Figure 6a, where the CV scans in the same buffer (pH 6.4) are displayed. Altogether, the experiments allowed the confirmation of redox couples for PYO and PQS centered in the pH 6.4 buffer at low potentials of −290 and 155 mV, respectively. In contrast, HQNO, PCH, and HHQ only underwent oxidation and/or reduction reactions without reversion. HQNO presented one oxidation peak at 325 mV (and a shoulder at 405 mV) and one unrelated reduction wave at −4 mV. PCH, on the other hand, gave two oxidation peaks at 594 mV and 806 mV, while HHQ showed an irreversible oxidation wave at a high potential of 1006 mV in the first CV cycle. The main oxidation reactions of PYO, PQS, HQNO, and the first peak of PCH had a peak difference of at least 170 mV each and could thus be clearly separated from each other. The second peak of PCH, however, partially overlapped with that of HHQ. Additionally, the peak of HHQ was located near the limit of the measurement range of the used SPE. Thus, it could be hindered by the hydrogen evolution from water electrolysis, also occurring at these high potentials. The reactions of PYO, PQS, and HQNO, on the other hand, occurred at low potentials where the peaks were generally well defined, even if the peak potential might vary because of occurring pH changes.

The limits of detection (LOD) and the corresponding optimal conditions on the SPEs were assessed by titrations of the substances in buffers of the entire pH range, in LB broth, as well as in *Pseudomonas*-selective medium (PMCN) using both electrochemical methods (CV and SWV). The measurements of PYO and HQNO turned out to work perfectly in media with the highest and most intense peak amplitudes and the lowest LODs compared to the measurements in other buffer systems. In addition, SWV proved to be the most sensitive detection method for both PYO as well as HQNO. For PYO, the LOD in CV (100 mV/s) and in buffers with pH 4.5 to 6.4 was approximately 4 µM, while by the use of SWV (amplitude 50 mV, frequency 5 Hz, step size 0.5 mV) in PMCN, a detection limit of 0.25 µM could be reached. The PYO content in ear secretions can be up to 2.7 µM [38], while it can reach 130 µM in the sputa of patients [39]. Thus, the LODs reported here are in perfect agreement with clinically relevant samples.

For HQNO, similar conclusions were drawn. Using SWV in a PMCN medium improved the detection limit to 2 µM compared to CV measurements in pH 7.4 buffer (15 µM). The titration of HQNO in PMCN and the calibration curve of the first peak are displayed in Figure 6b. On the other hand, testing PQS in media did not yield any improvements in terms of LOD. As described above, we found that the amplitude of the PQS peak decreased with elevated pH. Therefore, an acid pH of 3.8 proved to be a good assay condition for PQS, where an LOD of 4 µM could be achieved with SWV (Figure 6c). The tests of PQS in LB medium revealed comparable peak amplitudes and LOD as in acidic buffer systems. HQNO and PQS concentrations in culture supernatants of isolates were reported with 3–11 µM, and 3–5 µM [40,41], respectively, which also corresponds to the detection limits reached here.

However, in the cases of PCH and HHQ, measurements in media were difficult. Both analytes gave oxidation peaks at high potentials, where apparently, both LB broth and PMCN themselves showed strong oxidation signals that resulted in pronounced background noise. Thus, neither HHQ nor PCH could be discriminated from PMCN nor LB medium. The optimal parameters for measurements of HHQ and PCH turned out to be pH 6.4 and pH 7.4 buffers in either CV (100 mV/s) or SWV to achieve LODs of about 8 µM and 4 µM, respectively. While concentrations of HHQ in culture supernatant have been reported to be as high as 16.5 µM [40], the concentrations of PCH in *P. aeruginosa* samples are not known.

According to our results, the most promising metabolites for the detection of *P. aeruginosa* in a future sensor system are PYO, PQS, and HQNO. They can all be identified under similar conditions, e.g., in media in which *P. aeruginosa* is cultured. Moreover, they had clearly separated main peaks that occurred at low potentials where they were usually well defined. On the contrary, the peaks of PCH and HHQ partially overlapped during the measurements. Additionally, both were undetectable in media, and the peak of HHQ developed near the limit of the measurement range of the SPE. Detection of HHQ and PCH could thus be difficult under real conditions.

## 4. Conclusions

This analysis provides a comprehensive study for the potential use of the metabolites of *P. aeruginosa* as electrochemical biomarkers. While PYO, PQS, and HHQ had been previously studied electrochemically, we provide a more detailed overview and show several novel aspects of the analytes. In this study, we show—for the very first time—a general description of the voltammetric properties of PCH and HQNO. Both molecules have not been previously considered as additional markers for *P. aeruginosa* contaminations. According to our results, especially HQNO seems to be promising as a signal substance. It was identified here—for the first time—at low peak potentials and yielded additional oxidation and reduction peaks upon repeated cycling. This fact could be used as an additional marker to increase the specificity of the test system.

Extensive information on the pH dependence of all five metabolites was gained. We clearly show that HHQ could not be detected in highly acidic environments, whereas PCH split into one or two peaks depending on the pH of the sample. On the other hand, they could not be detected in growth media. In a biosensor system, approaches to adjusting the pH of samples could allow for the amplification of the signals. For example, in the case of PQS, more pronounced signals appeared at acidic pH values compared to neutral or basic samples. In the case of HHQ, raising the pH could also help to bring the peak out of the background.

All this information is crucial for predicting the corresponding signals in samples of diverse origins. Therefore, our structural description of the compounds is important for understanding and ultimately combating *P. aeruginosa* infections. We managed to provide a key step towards the development of a fully automated sensor system for quick in-situ analysis. Our characterization of the molecules can help define a precise, comprehensive profile specific to this opportunistic pathogen. Moreover, the reported concentrations of the preferred metabolites perfectly matched the detection limits achieved here. In summary, the SPEs are suitable for sensitive detection of the three metabolites. The use of SPEs could reduce the size of the instrument, decrease the sample volume, and allow for the construction of a portable system. Therefore, our study is relevant for the development of rapid detection systems for *P. aeruginosa* and thus for hospital hygiene and infection control.

## Data Availability

The data presented in this study are available in this article.

## Supplementary Materials

The following supporting information can be downloaded at: https://www.mdpi.com/article/10.3390/s22134694/s1, Figure S1: Changes in peak heights of PQS in CV with elevated scan rate; Figure S2: Scan rate influencing the peak height of HQNO during CV; Figure S3: Change in oxidation and reduction peak height of PYO as a function of the scan rate; Table S1: Cyclic voltammetry data of PYO after variation of scan rate.

## References

[1] Balabanova Y., Gilsdorf A., Buda S., Burger R., Eckmanns T., Gärtner B., Groß U., Haas W., Hamouda O., Hübner J. (2011). Communicable Diseases Prioritized for Surveillance and Epidemiological Research: Results of a Standardized Prioritization Procedure in Germany, 2011. PLoS ONE.

[2] CDC (2013). Antibiotic Resistance Threats in the United States, 2013.

[3] Neves P.R., McCulloch J.A., Mamizuka E.M., Lincopan N., Batt C.A., Tortorello M.-L. (2014). Aeruginosa. Encyclopedia of Food Microbiology.

[4] Planet P.J., Long S.S., Prober C.G., Fischer M. (2017). Pseudomonas aeruginosa. Principles and Practice of Pediatric Infectious Diseases.

[5] ECDC (2013). ECDC Surveillance Report. Point Prevalence Survey of Healthcare-Associated Infections and Antimicrobial Use in European Acute Care Hospitals, 2011–2012.

[6] Hidron A.I., Edwards J.R., Patel J., Horan T.C., Sievert D.M., Pollock D.A., Fridkin S.K. (2008). Antimicrobial-Resistant Pathogens Associated with Healthcare-Associated Infections: Annual Summary of Data Reported to the National Healthcare Safety Network at the Centers for Disease Control and Prevention, 2006–2007. Infect. Control Hosp. Epidemiol..

[7] Weiner L.M., Webb A.K., Limbago B., Dudeck M.A., Patel J., Kallen A.J., Edwards J.R., Sievert D.M. (2016). Antimicrobial-Resistant Pathogens Associated with Healthcare-Associated Infections: Summary of Data Reported to the National Healthcare Safety Network at the Centers for Disease Control and Prevention, 2011–2014. Infect. Control Hosp. Epidemiol..

[8] Weiner-Lastinger L.M., Abner S., Edwards J.R., Kallen A.J., Karlsson M., Magill S.S., Pollock D., See I., Soe M.M., Walters M.S. (2019). Antimicrobial-Resistant Pathogens Associated with Adult Healthcare-Associated Infections: Summary of Data Reported to the National Healthcare Safety Network, 2015–2017. Infect. Control Hosp. Epidemiol..

[9] Hossain Z., Motarjemi Y., Moy G., Todd E. (2014). Bacteria: *Pseudomonas*. Encyclopedia of Food Safety.

[10] Wu M., Guina T., Brittnacher M., Nguyen H., Eng J., Miller S.I. (2005). The *Pseudomonas aeruginosa* Proteome during Anaerobic Growth. J. Bacteriol..

[11] CDC (2019). Antibiotic Resistance Threats in the United States, 2019.

[12] Livermore D.M. (2002). Multiple Mechanisms of Antimicrobial Resistance in *Pseudomonas aeruginosa*: Our Worst Nightmare?. Clin. Infect. Dis..

[13] (2010). Meat and Meat Products-Enumeration of Presumptive Pseudomonas spp. (ISO 13720:2010).

[14] (2006). Water Quality-Detection and Enumeration of Pseudomonas aeruginosa-Method by Membrane Filtration (ISO 16266:2006).

[15] Matsuda K., Tsuji H., Asahara T., Kado Y., Nomoto K. (2007). Sensitive Quantitative Detection of Commensal Bacteria by rRNA-Targeted Reverse Transcription-PCR. Appl. Environ. Microbiol..

[16] Reynisson E., Lauzon H.L., Magnusson H., Hreggvidsson G.Ó., Marteinsson V.T. (2008). Rapid Quantitative Monitoring Method for the Fish Spoilage Bacteria *Pseudomonas*. J. Environ. Monit..

[17] Sandrin T.R., Goldstein J.E., Schumaker S. (2012). MALDI TOF MS Profiling of Bacteria at the Strain Level: A Review. Mass Spectrom. Rev..

[18] Singhal N., Kumar M., Kanaujia P.K., Virdi J.S. (2015). MALDI-TOF Mass Spectrometry: An Emerging Technology for Microbial Identification and Diagnosis. Front. Microbiol..

[19] Abbasian F., Ghafar-Zadeh E., Magierowski S. (2018). Microbiological Sensing Technologies: A Review. Bioengineering.

[20] Alatraktchi F.A., Svendsen W.E., Molin S. (2020). Electrochemical Detection of Pyocyanin as a Biomarker for *Pseudomonas aeruginosa*: A Focused Review. Sensors.

[21] Mavrodi D.V., Bonsall R.F., Delaney S.M., Soule M.J., Phillips G., Thomashow L.S. (2001). Functional Analysis of Genes for Biosynthesis of Pyocyanin and Phenazine-1-Carboxamide from *Pseudomonas aeruginosa* PAO1. J. Bacteriol..

[22] Reyes E.A.P., Bale M.J., Cannon W.H., Matsen J.M. (1981). Identification of *Pseudomonas aeruginosa* by Pyocyanin Production on Tech Agar. J. Clin. Microbiol..

[23] Nowroozi J., Akhavan Sepahi A., Rashnonejad A. (2012). Pyocyanine Biosynthetic Genes in Clinical and Environmental Isolates of *Pseudomonas aeruginosa* and Detection of Pyocyanine’s Antimicrobial Effects with or without Colloidal Silver Nanoparticles. Cell J..

[24] Sismaet H.J., Pinto A.J., Goluch E.D. (2017). Electrochemical Sensors for Identifying Pyocyanin Production in Clinical *Pseudomonas aeruginosa* Isolates. Biosens. Bioelectron..

[25] Price-Whelan A., Dietrich L.E.P., Newman D.K. (2006). Rethinking “secondary” Metabolism: Physiological Roles for Phenazine Antibiotics. Nat. Chem. Biol..

[26] Sismaet H.J., Banerjee A., McNish S., Choi Y., Torralba M., Lucas S., Chan A., Shanmugam V.K., Goluch E.D. (2016). Electrochemical Detection of *Pseudomonas* in Wound Exudate Samples from Patients with Chronic Wounds. Wound Repair Regen..

[27] Oziat J., Gougis M., Malliaras G.G., Mailley P. (2017). Electrochemical Characterizations of Four Main Redox–Metabolites of *Pseudomonas aeruginosa*. Electroanalysis.

[28] Zhou L., Glennon J.D., Luong J.H.T., Reen F.J., O’Gara F., McSweeney C., McGlacken G.P. (2011). Detection of the *Pseudomonas* Quinolone Signal (PQS) by Cyclic Voltammetry and Amperometry Using a Boron Doped Diamond Electrode. Chem. Commun..

[29] Buzid A., Shang F., Reen F.J., Muimhneacháin E.Ó., Clarke S.L., Zhou L., Luong J.H.T., O’Gara F., McGlacken G.P., Glennon J.D. (2016). Molecular Signature of *Pseudomonas aeruginosa* with Simultaneous Nanomolar Detection of Quorum Sensing Signaling Molecules at a Boron-Doped Diamond Electrode. Sci. Rep..

[30] Gethin G. (2007). The Significance of Surface PH in Chronic Wounds. Wounds UK.

[31] Tate S., MacGregor G., Davis M., Innes J.A., Greening A.P. (2002). Airways in Cystic Fibrosis Are Acidified: Detection by Exhaled Breath Condensate. Thorax.

[32] Häussler S., Becker T. (2008). The *Pseudomonas* Quinolone Signal (PQS) Balances Life and Death in *Pseudomonas aeruginosa* Populations. PLoS Pathog..

[33] Brandel J., Humbert N., Elhabiri M., Schalk I.J., Mislin G.L.A., Albrecht-Gary A.M. (2012). Pyochelin, a Siderophore of *Pseudomonas aeruginosa*: Physicochemical Characterization of the Iron(Iii), Copper(Ii) and Zinc(Ii) Complexes. Dalt. Trans..

[34] Bukelman O., Amara N., Mashiach R., Krief P., Meijler M.M., Alfonta L. (2009). Electrochemical Analysis of Quorum Sensing Inhibition. Chem. Commun..

[35] Morrison M.M., Seo E.T., Howie J.K., Sawyer D.T. (1978). Flavin Model Systems. 1. The Electrochemistry of 1-Hydroxyphenazine and Pyocyanine in Aprotic Solvents. J. Am. Chem. Soc..

[36] Sharp D., Gladstone P., Smith R.B., Forsythe S., Davis J. (2010). Approaching Intelligent Infection Diagnostics: Carbon Fibre Sensor for Electrochemical Pyocyanin Detection. Bioelectrochemistry.

[37] Elliott J., Simoska O., Karasik S., Shear J.B., Stevenson K.J. (2017). Transparent Carbon Ultramicroelectrode Arrays for the Electrochemical Detection of a Bacterial Warfare Toxin, Pyocyanin. Anal. Chem..

[38] Reimer Å., Edvaller B., Johansson B. (2000). Concentrations of the *Pseudomonas aeruginosa* Toxin Pyocyanin in Human Ear Secretions. Acta Oto-Laryngol. Suppl..

[39] Wilson R., Sykes D.A., Watson D., Rutman A., Taylor G.W., Cole P.J. (1988). Measurement of *Pseudomonas aeruginosa* Phenazine Pigments in Sputum and Assessment of Their Contribution to Sputum Sol Toxicity for Respiratory Epithelium. Infect. Immun..

[40] Lépine F., Milot S., Déziel E., He J., Rahme L.G. (2004). Electrospray/Mass Spectrometric Identification and Analysis of 4-Hydroxy-2-Alkylquinolines (HAQs) Produced by *Pseudomonas aeruginosa*. J. Am. Soc. Mass Spectrom..

[41] Ortori C.A., Dubern J.F., Chhabra S.R., Cámara M., Hardie K., Williams P., Barrett D.A. (2011). Simultaneous Quantitative Profiling of N-Acyl-l-Homoserine Lactone and 2-Alkyl-4(1H)-Quinolone Families of Quorum-Sensing Signaling Molecules Using LC-MS/MS. Anal. Bioanal. Chem..

