# Supplementary material for: Main Metabolites of Pseudomonas aeruginosa: A Study of Electrochemical Properties
Source: Sensors (Basel). 2022 Jun 22;22(13):4694. doi: 10.3390/s22134694 (PMC9269063; doi:10.3390/s22134694)
Supplement: Supplementary file 1 [file sensors-22-04694-s001.zip › sensors-1770428-supplementary.pdf]

Supplementary Material

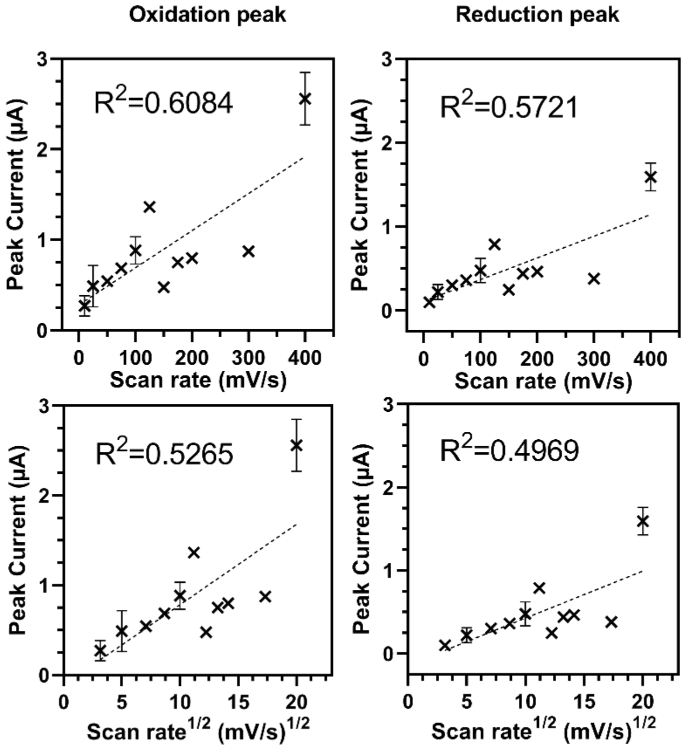

Figure S1. Changes in peak heights of 250 µM PQS in CV with elevated scan rate (10 to 400 mV/s, pH 6.4). Oxidation and reduction peak currents showed no linear dependency on the square root of the scan rate nor on the scan rate.

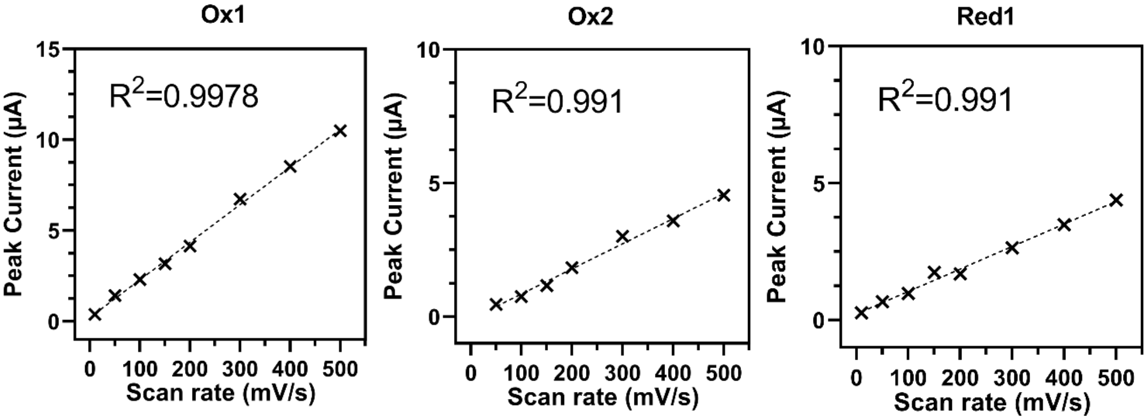

Figure S2. Scan rate influencing the peak height of HQNO during CV. The current responses of ox1 as well as those of the newly generated couple (ox2 and red1) vary linearly with the scan rate (10 mV to 500 mV/s, 125 µM HQNO in pH 7.4 buffer).

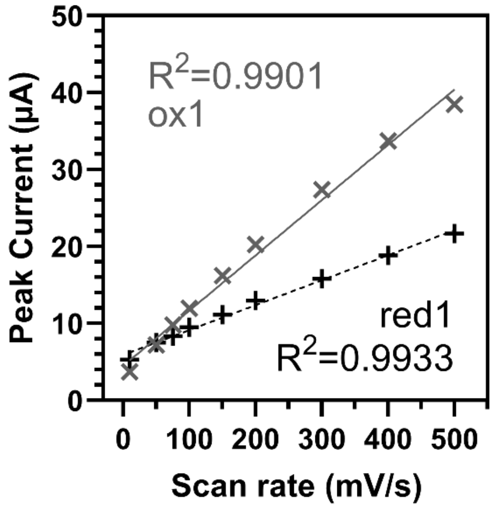

Figure S3. Change in oxidation and reduction peak height of PYO as a function of the scan rate. The currents of ox1 and red1 (125 µM PYO in buffer of pH 6.4) showed linear dependency on the scan rate (10 to 500 mV/s).

Table S1. Cyclic voltammetry data of 125 µM PYO in pH 6.4 buffer after varying the scan rate (10 to 500 mV/s). The ratio of the reduction peak current of red1 (I_{pc}) to the oxidation peak current of ox1 (I_{pa}) shows that PYO undergoes irreversible reduction at a slow scan rate of 10 mV/s. At faster scan rates (50 mV/s), reversibility improves, as confirmed by the presence of an increased oxidation peak. In addition, the peak-to-peak-separation (ΔE_p) varies with increased scan rate. Both results show a quasi-reversible redox system (ox1/ red1) of PYO.

Scan rate (mV/s)	I_{pc}/I_{pa}	ΔE_p (mV)
10	1.44	84.1
50	1.04	72
75	0.85	70
100	0.79	72
200	0.64	66
300	0.58	58
400	0.56	65.9
500	0.56	68
